# Metformin Enhances Doxycycline Efficacy Against *Pasteurella multocida*: Evidence from In Vitro, In Vivo, and Morphological Studies

**DOI:** 10.3390/microorganisms13081724

**Published:** 2025-07-23

**Authors:** Nansong Jiang, Weiwei Wang, Qizhang Liang, Qiuling Fu, Rongchang Liu, Guanghua Fu, Chunhe Wan, Longfei Cheng, Yu Huang, Hongmei Chen

**Affiliations:** 1 Research Center for Poultry Diseases, Institute of Animal Husbandry and Veterinary Medicine, Fujian Academy of Agricultural Sciences, Fuzhou 350013, China; nansongjiang@126.com (N.J.); wangweiweihn@163.com (W.W.); 11817043@zju.edu.cn (Q.L.); qiulingfu0822@163.com (Q.F.); liurongc@foxmail.com (R.L.); fuyuan163@163.com (G.F.); chunhewan@126.com (C.W.); lf396@139.com (L.C.); 2 Fujian Key Laboratory for Prevention and Control of Avian Diseases, Fuzhou 350013, China; 3 Fujian Industry Technology Innovation Research Academy of Livestock and Poultry Diseases Prevention & Control, Fuzhou 350013, China

**Keywords:** *Pasteurella multocida*, resistance, metformin, doxycycline, synergistic antibacterial activity

## Abstract

*Pasteurella multocida* (*Pm*) is a zoonotic pathogen that poses a significant threat to animal health and causes substantial economic losses, further aggravated by rising tetracycline resistance. To restore the efficacy of tetracyclines to *Pm*, we evaluated the synergistic antibacterial activity of doxycycline combined with metformin, an FDA-approved antidiabetic agent. Among several non-antibiotic adjuvant candidates, metformin exhibited the most potent in vitro synergy with doxycycline, especially against capsular serogroup A strain (*Pm*A). The combination demonstrated minimal cytotoxicity and hemolysis in both mammalian and avian cells and effectively inhibited resistance development under doxycycline pressure. At 50 mg/kg each, the combination of metformin and doxycycline significantly reduced mortality in mice and ducks acutely infected with *Pm*A (from 100% to 60%), decreased pulmonary bacterial burdens, and alleviated tissue inflammation and damage. Mechanistic validation confirmed that metformin enhances membrane permeability in *Pm* without compromising membrane integrity, dissipates membrane potential, increases intracellular doxycycline accumulation, and downregulates the transcription of the tetracycline efflux gene *tet*(B). Morphological analyses further revealed pronounced membrane deformation and possible leakage of intracellular contents. These findings highlight metformin as a potent, low-toxicity tetracycline adjuvant with cross-species efficacy, offering a promising therapeutic approach for managing tetracycline-resistant *Pm* infections.

## 1. Introduction

*Pasteurella multocida* (*Pm*) is a significant zoonotic pathogen capable of causing bacteremia in humans and a wide range of diseases in animals, including fowl cholera, swine pasteurellosis, and hemorrhagic septicemia in ruminants, leading to substantial economic losses in the livestock industry [1]. *Pm* is classified into at least five capsular serogroups (A, B, D, E, and F) and sixteen LPS types (1–16), while its multi-locus sequence typing (MLST) profiles exhibit considerable heterogeneity depending on host species and geographic origin [2]. Consequently, current vaccines often offer limited coverage and protection, making antimicrobial chemotherapy the primary approach for *Pm* infections [2,3]. Especially, *Pm* serotype A (*Pm*A) strains are the most frequently isolated, particularly from poultry, cattle, pigs, and rabbits [4], and are associated with severe respiratory infections and systemic bacteremia, frequently leading to acute mortality [2,4,5]. Our previous investigations demonstrated that the dominant clone A: L1: ST129 of *Pm* in Chinese poultry carries a high prevalence of resistance genes, particularly *sul2* (66.1%) and *tet*(B) (55.4%), conferring resistance to sulfonamides and tetracyclines, respectively [6]. A recent surveillance report revealed that over the past three years, tetracycline resistance of porcine *Pm* isolates in China has reached as high as 43.75% [7]. The presence of multiple resistance genes, especially those belonging to the tetracycline-resistance-associated *tet* family and its variants, are horizontally transferred via integrative conjugative elements, plasmids, and bacteriophages, further complicating the resistance landscape of *Pm* [2,6]. Given its clinical importance and growing antimicrobial resistance, *Pm* has been listed by the European Antimicrobial Resistance Surveillance network in Veterinary medicine as one of the top 11 veterinary pathogens of concern [8].

In the post-antibiotic era, there is an urgent need to develop effective and safe alternative therapies. One promising strategy involves repurposing existing drugs and combining conventional antibiotics with adjuvants that restore antimicrobial sensitivity [9,10,11]. Tetracycline antibiotics, including doxycycline and its derivatives, remain among the most widely used antimicrobials in veterinary clinical practice and agricultural settings due to their favorable tissue penetration, high oral bioavailability, and low cost [12]. As a result, the development of antimicrobial adjuvants targeting tetracyclines has gathered growing attention as a promising strategy to combat and delay antibiotic resistance [10,13,14]. Relevant studies have identified several FDA-approved non-antibiotic compounds as potential adjuvants capable of reversing tetracycline resistance, such as carprofen, benzydamine, metformin, benserazide, and loperamide [15,16,17,18]. Metformin, a first-line antidiabetic agent, has been shown to disrupt the bacterial membrane potential (Δψ), enhance antibiotic influx, and inhibit *tet*(A)-mediated efflux, thereby restoring tetracycline susceptibility [17,19]. Moreover, metformin has demonstrated synergistic effects with various antibiotics, including ampicillin, doxycycline, and chloramphenicol, and even combats tigecycline-resistant *Klebsiella pneumoniae* strains [19,20]. However, the efficacy and safety of antibiotic adjuvant combinations still require further validation across different host species, bacterial clones, and microbial populations [13,20,21]. It remains unclear whether metformin and other non-antibiotic adjuvants can enhance the therapeutic effectiveness of tetracyclines in treating infections caused by *Pm* and whether such combinations are safe for use in poultry. Resolving these issues is essential for advancing the clinical applicability of these therapeutic strategies.

In this study, *Pm*A was selected as the major target pathogen to compare the synergistic antibacterial effects of doxycycline in combination with five non-antibiotic compounds, including carprofen, benzydamine, metformin, benserazide, and loperamide. We further investigated the safety and in vivo therapeutic efficacy of the doxycycline–metformin combination and verified the mechanism by which metformin enhances the antibacterial activity of doxycycline against *Pm*. Specifically, the combination of metformin and doxycycline (i) exhibited the most potent synergistic effect among the tested compounds, (ii) significantly improved survival rates and reduced both inflammatory responses and bacterial burdens in *Pm*A-infected animals, and (iii) increased membrane permeability and enhanced intracellular accumulation of doxycycline. These findings further support the potential application of metformin as an effective tetracycline adjuvant, particularly against *Pm*A, and highlight its promise as an effective therapeutic strategy for veterinary use.

## 2. Materials and Methods

### 2.1. Bacterial Strains, Reagents, Cell Lines, and Culture Conditions

All *Pm* strains used in this study were described in our previous research [6,22], and detailed information is listed in Tables S1 and S2. Unless otherwise specified, all strains were cultured on tryptic soy agar or in tryptic soy broth (TSA/TSB; BD Difco^TM^, Franklin Lakes, NJ, USA) with 5% fetal bovine serum (FBS; Gibco, ThermoFisher, Waltham, MA, USA) at 37 °C for a minimum of 12 h. All chemical reagents and antibiotics (Table S3) used in this study were purchased from Macklin Biochemical Technology Co., Ltd. (Shanghai, China). Chinese hamster ovary (CHO) and chicken embryo fibroblast DF-1 cells were grown in Dulbecco’s Modified Eagle’s Medium (DMEM; Gibco, ThermoFisher, Waltham, MA, USA) supplemented with 10% heat-inactivated FBS, 1% (*w*/*v*) penicillin–streptomycin and 1% (*w*/*v*) sodium pyruvate. The cells were incubated at 37 °C in a humidified atmosphere with 5% CO_2_. Phosphate-buffered saline (PBS; pH 7.4) was used throughout the study for bacterial washing, dilution, and cell culture processing.

### 2.2. Minimum Inhibitory Concentration (MIC) Testing

According to the Clinical & Laboratory Standards Institute M100-Ed35 guidelines [23], the broth microdilution method was carried to access the antimicrobial susceptibility of *Pm* strains. Briefly, drugs were two-fold serially diluted in cation-adjusted Mueller–Hinton broth (CA-MHB; BD Difco^TM^, Franklin Lakes, NJ, USA) starting at 128 μg/mL, except for sulfadiazine, which started at 512 μg/mL. The initial concentrations of non-antibiotic compounds were selected based on pre-experimental MIC assessments. Each dilution was mixed with an equal volume of bacterial suspension (~1.5 × 10^6^ CFU/mL) in 96-well microtiter plates. *Escherichia coli* (ATCC^®^ 25922^TM^) served as the quality control strain. After 18 h incubation at 37 °C, the MIC values were defined as the lowest concentrations of antibiotics with no visible growth of bacteria.

### 2.3. Checkboard Assays

According to previous studies, the synergistic activity was assessed using the checkerboard microdilution method, and the corresponding fractional inhibitory concentration (FIC) index was calculated [17,18,24]. Briefly, antibiotics at a starting concentration of 4 × MIC and test compounds at 1 or 2 × MIC were two-fold serially diluted along the horizontal and vertical axes, respectively, in a 96-well plate containing CA-MHB, and then mixed with an equal volume of bacterial suspension (~1.5 × 10^6^ CFU/mL). After incubation at 37 °C for 18 h, the optical density (OD) at 600 nm of each well was measured using a microplate reader (Infinite^®^ 200 PRO, Tecan Group Ltd., Männedorf, Switzerland). The FIC index (FICI) was calculated using the formula:

$$\mathrm{FICI}\;=\;\frac{\left({\mathrm{MIC}}_{AB}/{\mathrm{MIC}}_{A}\right)}{\left({\mathrm{MIC}}_{BA}/{\mathrm{MIC}}_{B}\right)},$$

where MIC_A_ or MIC_B_ refer to the MIC of compound A or compound B when used alone, respectively, and MIC_AB_ or MIC_BA_ represent the MIC of compounds A or B when used in combination. Synergy was defined as FICI ≤ 0.5; no interaction was considered when 0.5 < FICI < 4; and antagonism was defined as FICI > 4.

### 2.4. Time-Dependent Killing Curve

Overnight culture of FCF83 was diluted 1:10,000 in CA-MHB and incubated at 37 °C for 4 h. Subsequently, cultures were treated with PBS, antibiotic agents at 1/2 or 1/4 or 1 × MIC concentrations, 8 mg/mL metformin, or their combinations. At 0, 2, 4, 8, 16, and 24 h post-treatment, 100 µL aliquots were collected, resuspended in PBS, and serially diluted. The dilutions were spotted onto TSA plates and incubated overnight at 37 °C. CFUs were then enumerated to assess bacterial survival rate.

### 2.5. Safety Assessment

The hemolytic activity of the doxycycline–metformin combination was evaluated based on previously described protocols with minor modifications [17]. Commercial sterile 6% rabbit or chicken erythrocytes (Solarbio Science & Technology Co., Ltd., Beijing, China) were co-incubated in equal volumes with the drug combinations at 37 °C for 1 h. After incubation, the absorbance of the supernatant at 576 nm was measured using a microplate reader. Triton X-100 (0.2%) was used as a positive control to induce complete hemolysis, while PBS served as the negative control. The hemolysis rate was calculated using the following formula:

$$Hemolysis=\frac{\left({OD}_{\mathrm{sample}}\;-\;{OD}_{\mathrm{PBS}}\right)}{\left({OD}_{\mathrm{Triton}}\;-\;{OD}_{\mathrm{PBS}}\right)}\times 100\%.$$

To evaluate cytotoxicity, CHO or DF-1 cells (1 × 10^4^ cells per well) were seeded into 96-well plates together with the combination of doxycycline and metformin. Cells were cultured in DMEM supplemented with 10% heat-inactivated FBS at 37 °C for 24 h. Cell viability was then assessed using the CCK-8 assay kit (Solarbio Science & Technology Co., Ltd., Beijing, China) according to the manufacturer’s instructions. The viability rate was calculated using the following formula:

$$Cell\;viablity\left(\%\right)=\frac{\left({OD}_{\mathrm{treatment}}\;-\;{OD}_{\mathrm{blank}}\right)}{\left({OD}_{\mathrm{control}}\;-\;{OD}_{\mathrm{blank}}\right)}\times 100\%.$$

### 2.6. Development of Antibiotic Resistance

This experiment was performed with slight modifications based on a previously described method [25]. Exponentially growing FCF83 cultures were diluted 1:1000 into CA-MHB medium containing either doxycycline (8 μg/mL), metformin (16 mg/mL), or a combination of both. Cultures were incubated at 37 °C for 24 h. The MIC was determined daily by broth microdilution in 96-well plates. The cultures were then diluted into fresh medium with the same drug concentrations for the next passage. This process was repeated daily for 40 consecutive days. Fold changes in doxycycline MICs were calculated relative to the initial MIC.

### 2.7. Animal Ethics and Husbandry

All animal experiments followed the Guide for the Care and Use of Laboratory Animals (National Research Council, 8th edition). The procedure was approved by the Research Ethics Committee of the Institute of Animal Husbandry and Veterinary Science of Fujian Academy of Agricultural Sciences (Approval No. MYLISC2024-019, Approval Date 19 December 2024). The number of animals used in this study was determined based on ethical considerations and preliminary assessments of *Pm* virulence. The sample size was sufficient to compare therapeutic outcomes of the drug combination in mammalian and avian models of infection. In compliance with the 3R principles (Replacement, Reduction, and Refinement), unnecessary animal use was minimized. All animals were confirmed to be clinically healthy and free of specific pathogens prior to experimentation. Mice and ducks were housed in standard plastic cages with wood shavings and in well-ventilated poultry enclosures, respectively. Environmental conditions were maintained at 22 ± 2 °C with approximately 50% relative humidity and a 12-h light/dark cycle. Animals were provided with unrestricted access to food and water. Given the acute nature of *Pm* infection, animals were monitored every 2 h for clinical signs. Humane endpoints included severe clinical symptoms, upon which euthanasia was performed via intraperitoneal injection of chloral hydrate. To avoid interference with the tested drugs, no additional analgesics or anesthetics were administered during the experiments.

### 2.8. In Vivo Evaluation of Combination Therapy

To evaluate the in vivo efficacy of doxycycline combined with metformin, mouse and duck infection models of *Pm* were established. Female ICR mice (6–8 weeks old, 25–30 g, n = 40) were obtained from the Experimental Animal Center of Fujian Agriculture and Forestry University. Cherry Valley ducks (30–45 days old, 2.5–3.0 kg, n = 40) were purchased from a local farm. All animals were randomly divided into four groups (n = 10 per group). Mice and ducks were intraperitoneally and intramuscularly injected, respectively, with 100 μL of a lethal dose of FCF83 strain (45 CFU). Four hours post-infection, the animals were treated with 100 μL of the following: doxycycline (50 mg/kg), metformin (50 mg/kg), a combination of doxycycline and metformin (50 mg/kg + 50 mg/kg), or PBS as control. The bacterial challenge dose and drug administration regimen used in the animal experiments were determined based on our previous study [22], relevant literature [17,26,27,28], preliminary in vitro assays, and pilot in vivo trials. Survival status was assessed at 2 h intervals over a 7-day observation period. Lung tissues from mice or ducks that succumbed 18–24 h post-infection were aseptically collected and homogenized. The homogenates were serially diluted and plated on TSA plates, followed by incubation at 37 °C for 24 h. Bacterial loads were quantified by CFU.

### 2.9. Histopathological Examination

To evaluate histopathological changes, the hearts, livers, lungs, and kidneys were collected from both surviving and deceased mice and ducks in each group. The tissues were fixed in 4% paraformaldehyde, dehydrated, embedded in paraffin, and sectioned into 4 μm thick slices. Sections were mounted on microscope slides and stained with hematoxylin and eosin (H&E) following standard protocols. Histopathological alterations were assessed under a light microscope.

### 2.10. One-Step In Vitro Growth Curve

To preliminarily assess the effect of different concentrations of metformin on the growth of *Pm*, mid-log phase bacterial cultures were diluted in CA-MHB to an initial OD600 of 0.1. Metformin was added at final concentrations ranging from 0 to 16 mg/mL. The cultures were incubated at 37 °C with shaking at 200 rpm. Bacterial growth was monitored by measuring OD600 every 30 min over a 24 h period using a microplate reader.

### 2.11. Detection of Membrane Permeability and Integrity, Proton Motive Force (PMF), and Drug Uptake

Overnight cultures of FCF83 strain were collected, washed twice with 5 mM 4-(2-hydroxyethyl)-1-piperazineethanesulfonic acid (HEPES) buffer containing 5 mM glucose, and adjusted to an OD_600_ of 0.5 for all fluorescence-based assays.

Outer membrane permeability was assessed using the hydrophobic fluorescent probe 1-N-phenylnaphthylamine (NPN, 10 μM), which exhibits enhanced fluorescence upon entering the hydrophobic environment of the bacterial outer membrane when its integrity is compromised. After pre-incubation of bacterial suspensions with metformin (0–8 mg/mL, at 37 °C for 30 min), NPN was added and incubated for another 30 min. Fluorescence was measured with excitation at 350 nm and emission at 420 nm.

Membrane integrity was assessed using propidium iodide (PI, 10 nM). After metformin pre-treatment (0–8 mg/mL, at 37 °C for 30 min), PI was added to the bacterial suspensions and incubated for 30 min at 37 °C in the dark. Fluorescence was detected at 535 nm excitation and 617 nm emission. Increased fluorescence indicates compromised membrane integrity.

PMF measurement was divided into two components: (i) To assess membrane potential, 3,3-dipropylthiadicarbocyanine iodide (DiSC_3_(5), 0.5 μM) was added to the bacterial suspension to allow fluorescence quenching upon membrane binding. When membrane depolarization occurs, the dye is released into the extracellular environment, resulting in increased fluorescence. After 30 min of incubation, metformin was added at final concentrations of 0 to 8 mg/mL, and fluorescence was measured after another 30 min using excitation at 350 nm and emission at 420 nm. (ii) To assess intracellular pH, the pH-sensitive fluorescent dye 2′,7′-Bis-(2-carboxyethyl)-5-(and-6)-carboxyfluorescein acetoxymethyl ester (BCECF-AM, 20 μM) was added. Its fluorescence intensity increases with rising intracellular pH. Cells were incubated with the dye for 30 min, followed by treatment with metformin (0–8 mg/mL, at 37 °C for 30 min). Fluorescence was then recorded using excitation at 500 nm and emission at 522 nm.

Doxycycline uptake assay was performed based on previously described methods [16,18], with minor modifications. Briefly, sub-MIC concentrations of doxycycline (8 μg/mL) were added alone or in combination with metformin (0–8 mg/mL) to bacterial suspensions and incubated at 37 °C for 30 min. The reaction was immediately quenched on ice, and cells were washed thoroughly with pre-chilled PBS to remove extracellular doxycycline, then resuspended in PBS. Intracellular fluorescence was measured promptly using a microplate reader with excitation at 405 nm and emission at 535 nm.

### 2.12. Quantitative Real-Time PCR (qRT-PCR) Assay

Overnight cultures of FCF83 were diluted 1:100 into fresh TSB containing metformin (0–8 mg/mL) and incubated at 37 °C until mid-log phase. Total RNA was extracted using the RNAprep Pure Animal/Cell/Bacteria Kit (Tiangen Biotech Co., Ltd., Beijing, China), and complementary DNA (cDNA) was synthesized with the PrimeScript RT reagent kit (Takara Biomedical Technology Co., Ltd., Beijing, China). qRT-PCR was performed using TB Green Premix Ex Taq II (Takara, Kusatsu, Japan) on a CFX96 RT-PCR System (Bio-Rad Laboratories, Inc., Berkeley, CA, USA) with primers specific to the chromosomal encoded *tet*(B) gene (forward: TTCAAGTGCGCTTTGGATGC; reverse: CGTTGAGAAGCTGAGGTGGT) [29]. The thermal profile was 95 °C for 30 s, followed by 40 cycles of 95 °C for 5 s and 60 °C for 34 s. Relative expression levels were calculated using the 2^−ΔΔCt^ method.

### 2.13. Bacterial Morphological Analysis

Mid-log phase cultures of FCF83 were collected and treated with 8 mg/mL metformin for 1 h. After incubation, cells were washed with PBS and pelleted by centrifugation. Bacterial pellets were fixed overnight at 4 °C with 2.5% glutaraldehyde, followed by post-fixation with 1% osmium tetroxide for 1 h. Samples were then dehydrated through a graded ethanol series (30% to 100%). For transmission electron microscopy (TEM), samples were embedded in epoxy resin. Ultrathin sections (70–90 nm) were prepared using an ultramicrotome, stained with 2% uranyl acetate and lead citrate, and examined using a transmission electron microscope (H-7650, Hitachi, Tokyo, Japan) at an accelerating voltage of 80 kV. For scanning electron microscopy (SEM), samples were subjected to critical point drying using CO_2_, followed by sputter coating with gold. Imaging was performed using a scanning electron microscope (SU8100, Hitachi, Tokyo, Japan) at an accelerating voltage of 3.0 kV.

### 2.14. Statistical Analyses

All data are presented as means ± standard deviations (SDs) and were subjected to analysis using nonparametric one-way ANOVA followed in GraphPad Prism v10.3.1 (GraphPad Software Inc., La Jolla, CA, USA). Survival curves were analyzed using Kaplan–Meier analysis in GraphPad Prism. Statistical significance was determined at a *p*-value of <0.05 (*), <0.01 (**), <0.001 (***), or <0.0001 (****), while *p*-values exceeding 0.05 were regarded as not significant (ns).

## 3. Results

### 3.1. In Vitro Synergistic Antibacterial Activity of Drug Combinations Against Pm

**Figure 1:**
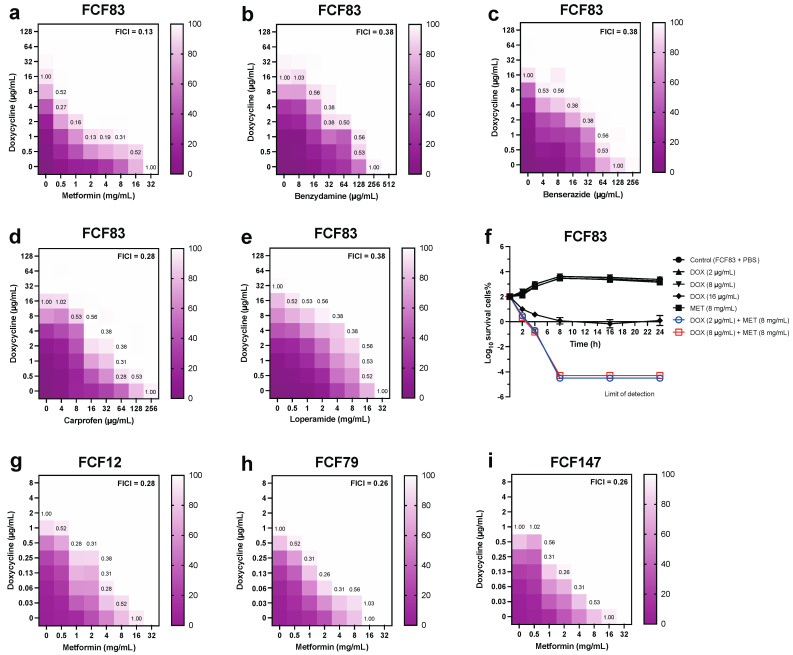
Checkerboard and time–kill analyses evaluating the synergistic antibacterial effects of doxycycline (DOX) combined with five non-antibiotic adjuvants against *Pm*. (**a**–**e**) Heatmaps show the FICI and growth inhibition (%) of DOX combined with metformin, benzydamine, benserazide, carprofen, and loperamide, respectively. Synergistic interactions (FICI ≤ 0.5) were observed for all five compounds, with metformin showing the strongest synergy (FICI = 0.13). Gradient bars indicate the percentage of bacterial growth inhibition; (**f**) Time–kill curves demonstrate the enhanced bactericidal activity of DOX when combined with metformin (8 mg/mL) at different DOX concentrations (2, 8, and 16 μg/mL) over 24 h; (**g**–**i**) Heatmaps show that DOX–metformin also exerts synergy against other *Pm* strains including FCF12 (serogroup D), FCF79 (serogroup F), and FCF147 (capsule-deficient mutant), with strongest activity against the serogroup A strain FCF83. All data represent results from three independent experiments.

To investigate potential tetracycline adjuvants for combatting multidrug-resistant Pm, we selected the *tet*(B)-harboring strain FCF83 as the representative strain for in vitro synergy evaluation. Checkerboard assays revealed that all five FDA-approved non-antibiotic compounds—metformin, benzydamine, carprofen, benserazide, and loperamide—enhanced doxycycline’s inhibitory activity to varying extents (Figure 1a–e). Notably, metformin exhibited the most potent synergistic effect, with a lowest FICI of 0.13, indicating strong synergy (Figure 1a). The synergistic effect of metformin was most pronounced at 1–4 μg/mL doxycycline combined with 0.5–8 mg/mL metformin. This was further supported by time–kill kinetics, where the combination of metformin and doxycycline achieved more rapid and sustained bacterial killing compared to either monotherapy (Figure 1f). Specifically, when combined with 8 mg/mL metformin, 2 μg/mL doxycycline achieved an inhibitory effect comparable to that of 8 μg/mL doxycycline alone. In addition to metformin, other non-antibiotic adjuvants such as benzydamine and carprofen also exhibited synergistic effects with doxycycline (FICI = 0.28–0.38), while benserazide and loperamide showed weaker synergy or no interaction in certain concentration ranges.

To assess strain- and serotype-specific responses, we extended synergy testing to additional isolates, including FCF12 (serotype D), FCF79 (serotype F), and the capsule-deficient mutant FCF147. The metformin–doxycycline combination maintained synergistic activity across all strains tested (FICI = 0.26–0.28), although the most pronounced effect remained against serotype A (FICI = 0.13) (Figure 1g–i). Broader screening of metformin combined with other antibiotic classes revealed selective synergism (Figure S1a–h): metformin potentiated the activity of tetracycline (FICI = 0.13) and erythromycin (FICI = 0.5), while displaying additive or indifferent interactions with ampicillin, chloramphenicol, sulfadiazine, and ciprofloxacin. However, striking antagonism was observed when metformin was combined with aminoglycosides such as kanamycin (FICI > 4.5) and streptomycin (FICI > 4.25). Consistent with checkerboard results, time–kill assays further validated the strong synergy between metformin and tetracyclines against Pm (Figure S1i), suggesting that the beneficial effects of metformin are largely tetracycline-specific.

### 3.2. In Vivo Therapeutic Efficacy of Combined Metformin and Doxycycline Against Pm Infection

Before evaluating the therapeutic efficacy of metformin and doxycycline against *Pm* infection in animals, we repeated a preliminary safety assessment based on previous studies [17]. Cell viability assays revealed that combinations of metformin (0–8 mg/mL) and doxycycline (0–32 μg/mL) exerted negligible cytotoxic effects on CHO and DF-1 cells, maintaining survival rates above 97% (Figure S2a,b). Similarly, hemolysis rates remained under 2% in both rabbit and chicken erythrocytes following treatment with the combination, suggesting minimal hemolytic activity (Figure S2c,d). Furthermore, compared to doxycycline alone, the combination with metformin effectively prevented the emergence of resistance, further supporting its utility in preserving antibiotic efficacy (Figure S2e).

**Figure 2:**
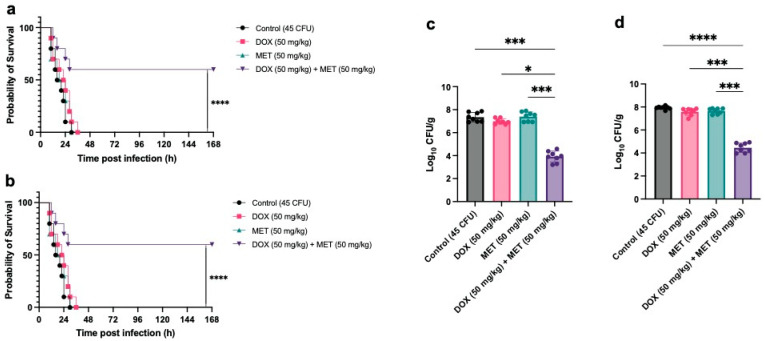
In vivo therapeutic efficacy of doxycycline–metformin combination in murine and avian models of Pm infection. (**a**,**b**) Survival curves of ICR mice (**a**) and Cherry Valley ducks (**b**) infected with a lethal dose of *Pm*A (45 CFU) and treated with doxycycline (DOX, 50 mg/kg), metformin (MET, 50 mg/kg), their combination (DOX + MET), or PBS (Control). The combination therapy significantly improved survival rates compared to monotherapy or control (Kaplan–Meier analysis, *p* < 0.001 (***)); (**c**,**d**) Pulmonary bacterial loads (log_10_ CFU/g) in deceased mice (**c**) and ducks (**d**) at 18–24 h post-infection. The DOX + MET group showed markedly reduced bacterial burdens. Statistical significance was determined using nonparametric one-way ANOVA: *p* < 0.05 (*), *p* < 0.001 (***), *p* < 0.0001 (****).

We established *Pm*A infection models in ICR mice and Cherry Valley ducks to evaluate the in vivo therapeutic effects of doxycycline, metformin, and their combination. Due to the high virulence of *Pm*A, administration of a low dose (45 CFU) led to 100% mortality within 36 h in both animal models. Monotherapy with either doxycycline or metformin showed no significant improvement in survival. However, combination therapy markedly reduced mortality, lowering the death rate from 100% to 60% in acutely infected animals (Figure 2a,b). Bacterial load analysis further revealed that the combination significantly decreased pulmonary bacterial burdens in mice and ducks that succumbed within 18–24 h post-infection (Figure 2c,d), supporting the in vivo synergistic efficacy of the metformin–doxycycline treatment.

**Figure 3:**
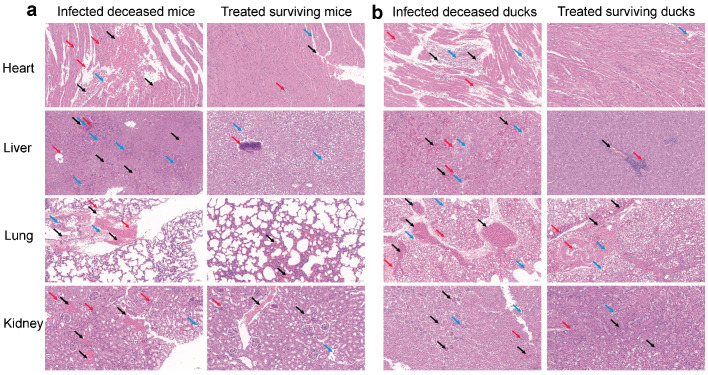
Histopathological analysis of tissues from animal models treated with doxycycline–metformin combination following Pm infection (H&E staining; scale bar = 50 μm). (**a**) Representative histological sections of heart, liver, lung, and kidney from mice that succumbed to infection after monotherapy with doxycycline (50 mg/kg) or metformin (50 mg/kg), compared to those that survived combination treatment (50 mg/kg + 50 mg/kg); (**b**) Corresponding tissue sections from Cherry Valley ducks under the same treatment groups. Black arrows indicate vascular congestion and erythrocyte infiltration; red arrows indicate lymphocyte infiltration; blue arrows denote cellular degeneration and focal necrosis. Tissues from animals treated with the combination therapy exhibited substantially alleviated pathological damage compared to monotherapy groups.

Histopathological analysis was further conducted on tissues from infected dead and drug-treated surviving mice and Cherry Valley ducks. Infected dead mice showed extensive erythrocyte leakage, lymphocyte infiltration, structural disruption, and focal necrosis in the heart, liver, lungs, and kidneys (Figure 3a). In contrast, organs from mice that survived combined metformin and doxycycline treatment exhibited only mild inflammatory cell aggregation and retained relatively intact tissue architecture with significantly alleviated inflammation. Notably, while residual pulmonary lesions were still evident in some surviving ducks (Figure 3b), the overall histological improvements underscore the therapeutic benefit of the combined regimen.

Collectively, these in vivo findings demonstrate that metformin significantly enhances the therapeutic efficacy of doxycycline against *Pm*A infection across two animal models, while exhibiting minimal toxicity and preserving tissue integrity. These results support the potential application of this combination in veterinary medicine for combating drug-resistant *Pm* infections.

### 3.3. Mechanistic Verification of the Synergistic Effect Between Metformin and Doxycycline Against Pm

Previous studies have demonstrated that metformin can destabilize the bacterial outer membrane, thereby enhancing the intracellular influx and accumulation of tetracycline-class antibiotics [17,19,28]. To validate the mechanism underlying the synergistic antibacterial effect of metformin and doxycycline against *Pm*, we conducted a series of confirmatory and complementary assays, focusing on membrane homeostasis, antibiotic uptake, and gene expression.

**Figure 4:**
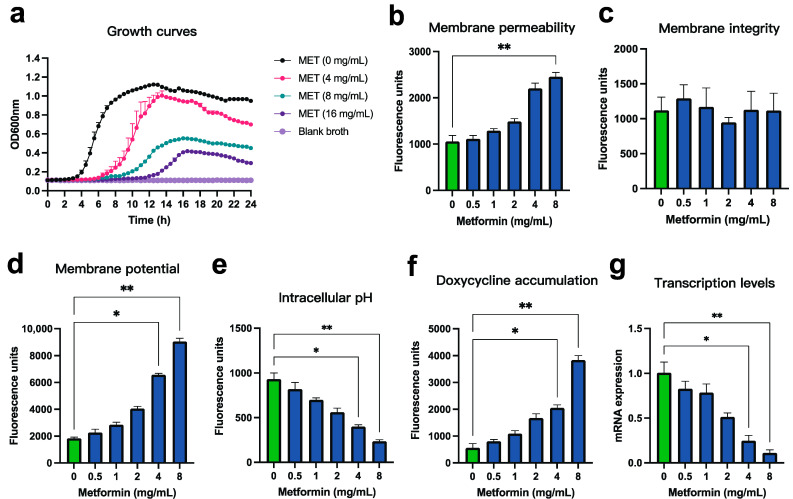
Mechanistic verification analysis of the synergistic antibacterial effect of metformin and doxycycline against *Pm*. (**a**) One-step in vitro growth curves of *Pm* strain FCF83 treated with increasing concentrations of metformin (0–16 mg/mL); (**b**) Outer membrane permeability assay using the hydrophobic fluorescent probe NPN. Increased fluorescence indicates enhanced permeability upon treatment with metformin (0–8 mg/mL); (**c**) Membrane integrity analysis using the fluorescent dye PI showed no significant differences in fluorescence intensity among the treatment groups; (**d**) Membrane potential (Δψ) measurements using DiSC_3_(5), which exhibits increased fluorescence upon depolarization as the dye is released into the extracellular environment; (**e**) Intracellular pH (ΔpH) detection using BCECF-AM, a pH-sensitive dye whose fluorescence increases with higher cytoplasmic pH; (**f**) Doxycycline accumulation assay in *Pm* cells based on its intrinsic fluorescence, indicating intracellular uptake under metformin treatment (0–8 mg/mL); (**g**) Relative transcription levels of the tetracycline efflux gene *tet*(B) under metformin treatment (0–8 mg/mL) as determined by qRT-PCR. All experiments were performed in triplicate. The green bars represent the negative control group without metformin, while the blue bars represent experimental groups treated with different concentrations of metformin. Data are presented as mean ± SD (n = 3). Statistical significance was determined by nonparametric one-way ANOVA: *p* < 0.05 (*), <0.01 (**).

We first examined whether metformin directly inhibits *Pm* growth. One-step growth curve analysis revealed that even at high concentrations (up to 16 mg/mL), metformin exhibited only mild growth-inhibitory effects on *Pm* strain FCF83, suggesting its antibacterial potency is limited when used alone (Figure 4a). This reinforces the idea that its primary value lies in synergizing with antibiotics rather than acting independently. To assess changes in outer membrane permeability, we employed the hydrophobic fluorescent probe NPN, which fluoresces upon entering the hydrophobic core of a compromised outer membrane. A dose-dependent increase in fluorescence was observed following metformin treatment (0–8 mg/mL), indicating significantly increased outer membrane permeability (Figure 4b). In contrast, PI staining showed no significant difference in fluorescence intensity across the same concentration range (Figure 4c), suggesting that metformin does not disrupt overall membrane integrity. We next evaluated the effect of metformin on bacterial PMF, a critical energy-generating system that drives antibiotic efflux and nutrient uptake. Using the voltage-sensitive dye DiSC_3_(5), we observed that metformin caused marked membrane depolarization, evidenced by increased fluorescence due to dye release into the extracellular space (Figure 4d). Simultaneously, BCECF-AM staining revealed a reduction in intracellular pH, indicating a compensatory increase in ΔpH as membrane potential (Δψ) decreased (Figure 4e). To determine whether these physiological alterations affect antibiotic accumulation, we leveraged the intrinsic fluorescence of doxycycline. Metformin co-treatment significantly increased the intracellular fluorescence signal in a dose-dependent manner, suggesting enhanced antibiotic uptake (Figure 4f). In parallel, qRT-PCR analysis showed that metformin suppressed the transcription of *tet*(B), a key tetracycline efflux pump gene, further contributing to intracellular drug retention (Figure 4g). Taken together, these findings demonstrate that metformin enhances the intracellular accumulation of doxycycline in Pm by increasing membrane permeability and disrupting energetic potential, without compromising membrane integrity. Additionally, metformin downregulates the transcription of the tetracycline efflux pump gene *tet*(B), thereby potentiating doxycycline’s antibacterial activity.

**Figure 5:**
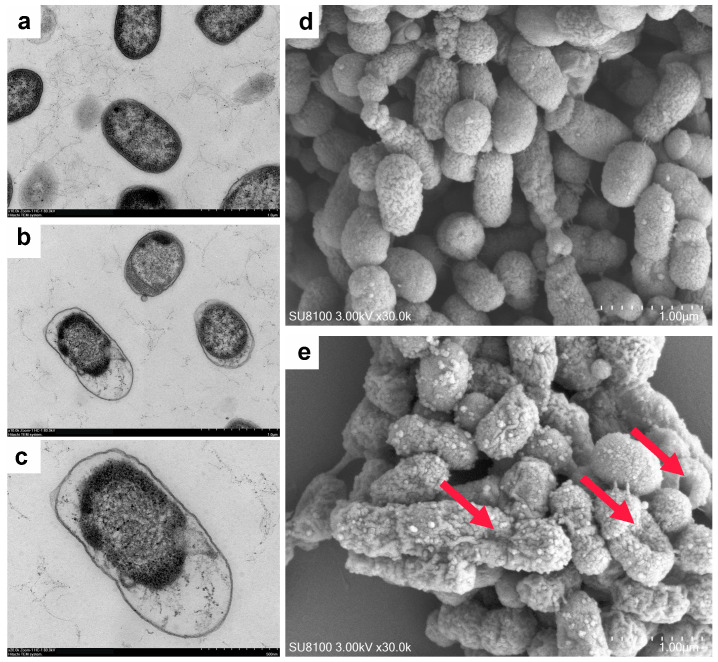
Morphological analysis of *Pm* under metformin treatment (8 mg/mL). (**a**) TEM image of FCF83 under normal culture conditions, showing intact membrane structure and dense cytoplasm (scale bar = 1 μm); (**b**) TEM image of FCF83 treated with metformin, displaying disrupted reduced cytoplasmic density (scale bar = 1 μm); (**c**) Higher-magnification TEM image revealing vacuole-like structures and potential leakage of intracellular contents (scale bar = 500 nm); (**d**) SEM image of untreated FCF83 cells with smooth, intact surfaces and regular morphology (scale bar = 1 μm); (**e**) SEM image of metformin-treated FCF83 cells showing pronounced surface wrinkling, collapse, and structural distortion (scale bar = 1 μm); red arrows indicate typical membrane depressions and folds.

We further examined the morphological changes of *Pm* under metformin treatment. TEM revealed that, compared with the structurally intact and cytoplasm-dense morphology of untreated FCF83 cells (Figure 5a), metformin-treated bacteria exhibited cytoplasmic sparsity and vacuole-like structures, suggesting potential leakage of intracellular contents (Figure 5b,c). SEM showed that, in contrast to the smooth and regularly shaped surface of untreated FCF83 cells (Figure 5d), metformin-treated cells displayed pronounced surface wrinkling and collapse, indicating varying degrees of morphological distortion (Figure 5e). These TEM and SEM observations suggest that metformin may disturb the outer membrane structure or alter membrane lipid composition, thereby increasing membrane permeability and leading to instability and surface deformation—consistent with its proposed mechanism of destabilizing bacterial membranes and enhancing antibiotic synergy. Together, SEM and TEM analyses support that metformin disrupts bacterial membrane stability and permeability, consistent with its role in enhancing antibiotic synergy.

## 4. Discussion

The growing prevalence of antimicrobial resistance in veterinary pathogens has rendered the treatment of *Pm*, particularly *Pm*A, increasingly challenging. This is largely due to its high virulence, widespread distribution, and frequent acquisition of multidrug resistance traits [8]. The high prevalence of tetracycline resistance gene *tet*(B) among *Pm* isolates in China raises concerns regarding the clinical efficacy of tetracycline therapy [6]. Repurposing existing drugs is considered an effective strategy for overcoming resistance [10,21], as evidenced by compounds like epigallocatechin gallate, which can restore the activity of fluoroquinolones against resistant *Pm* strains [30]. 

Among the five FDA-approved non-antibiotic compounds—carprofen, benzydamine, metformin, benserazide, and loperamide—that have been shown to reverse tetracycline resistance in certain bacterial strains [15,16,17,18], metformin exhibited the most potent synergistic effect when combined with doxycycline, both in vitro and in vivo. This finding is consistent with previous reports demonstrating the ability of metformin to restore tetracycline activity in resistant strains, including those tigecycline-resistant variants carrying the *tmexCD1-toprJ1* resistance cluster [17,19,20,27,28]. Notably, metformin displayed antagonism with aminoglycosides such as kanamycin and streptomycin (Figure S1), likely due to its disruption of membrane potential, the stability of which is essential for active aminoglycoside uptake [31,32]. In contrast, erythromycin was the only macrolide showing synergy with metformin, a phenomenon possibly attributable to species-specific factors. For instance, previous studies have shown metformin synergizes with rifampicin, ampicillin, or levofloxacin in various Gram-positive and Gram-negative bacteria [20], while other reports suggest it may not enhance—and could even antagonize—the activity of ampicillin or ciprofloxacin in *Acinetobacter baumannii* [28]. Similarly, another study reported that metformin not only exhibited antagonistic interactions when combined with β-lactams or aminoglycosides but also showed divergent effects—either synergistic or antagonistic—when used in combination with ciprofloxacin against *Klebsiella pneumoniae* strains carrying different resistance genes [19]. These findings further underscore metformin’s potential as an antibiotic adjuvant while highlighting the need for comprehensive evaluation of its efficacy in combination with various antibiotics across different bacterial species.

Safety is a critical consideration for any antimicrobial adjuvant. Metformin, a well-established and widely used antidiabetic agent, has demonstrated an excellent safety profile [33]. In this study, we expanded safety evaluations to include avian DF-1 fibroblasts and chicken erythrocytes (Figure S2), due to the host preference of *Pm*A in poultry [6]. The doxycycline–metformin combination showed minimal cytotoxicity to mammalian and avian cells and <5% hemolysis, which is considered biologically safe [34]. Moreover, one study demonstrated that the combination of metformin with various antibiotics did not affect body weight or blood glucose levels in mice [20], further underscoring its safety in these aspects. However, it is important to note that the primary adverse effect of metformin in mammals is lactic acidosis, which results from inhibited hepatic gluconeogenesis from lactate, leading to lactate accumulation [35]. It remains possible that similar effects could occur when metformin is combined with doxycycline. Therefore, further in vivo investigations are warranted to determine whether this combination can avoid such common toxic side effects.

Importantly, metformin suppressed the emergence of doxycycline resistance during prolonged exposure (Figure S2), echoing earlier findings in *Escherichia coli* [17,28]. This may be attributed not only to its intrinsic antibacterial activity but also to its impact on bacterial physiology, such as inhibition of biofilm formation in *Acinetobacter baumannii* [28] and quorum-sensing suppression in *Pseudomonas aeruginosa* [36]. Nonetheless, a recent study reported that metformin could upregulate efflux systems like AcrAB-TolC and EmrKY-TolC while suppressing iron transporters in *Escherichia coli*, potentially contributing to multidrug resistance [37]. These contrasting effects warrant more systematic studies to determine the long-term consequences of metformin use as an antibiotic adjuvant.

In animal infection models, the combination therapy significantly reduced the mortality of mice and ducks challenged with a lethal dose of *Pm*A, from 100% to approximately 60%, and also reduced pulmonary bacterial loads (Figure 2). However, this effect was less pronounced than that observed in similar studies involving *E. coli* or *Shigella* spp. [17,20], likely due to the acute tissue damage caused by the high virulence of the *Pm*A strain. Histopathological analysis supported this hypothesis, as treated animals still showed mild tissue damage and inflammation despite improved survival (Figure 3). Moreover, metformin is known to modulate host immune responses [17]. It can lower infection-induced erythrocyte sedimentation rate and white blood cell count [20], inhibit intracellular growth of *Mycobacterium tuberculosis* via adenosine monophosphate-activated protein kinase (AMPK) activation [38], and enhance macrophage antibacterial activity against *Mycobacterium avium* [39]. These host-directed effects may further contribute to its therapeutic benefit. However, factors such as gut microbiota composition have been shown to influence metformin–antibiotic interactions in vivo [40]. Future studies are needed to evaluate pharmacokinetics, bioavailability, and microbiome impacts of such combinations in veterinary species.

Previous studies have extensively explored the mechanisms by which metformin reverses tetracycline resistance [17]. In this study, we replicated those findings (Figure 4) and further validated them with morphological analyses (Figure 5). Growth curve assays showed that metformin alone exhibited a modest inhibitory effect on *Pm* at high concentrations, consistent with reports suggesting reduced infection rates among diabetic patients receiving metformin, thereby implying its potential intrinsic antibacterial activity [41,42]. Our results confirmed that metformin enhances the permeability of the outer membrane, dissipates membrane potential, and compensatorily increases the pH gradient, which collectively promote doxycycline uptake. In parallel, *tet*(B) expression was downregulated, thereby reducing antibiotic efflux. SEM analysis revealed that metformin disrupted membrane stability and induced surface deformation, likely by altering membrane lipid composition—supporting its proposed mechanism of destabilizing bacterial membranes to potentiate antibiotic action. TEM images further corroborated the increase in membrane permeability, showing cytoplasmic sparsity and vacuole-like structures. We speculate that PMF disruption may impair membrane function and cause leakage of intracellular contents, leading to the formation of low-density regions. Interestingly, a separate study reported that metformin inhibited minocycline-resistant *A. baumannii* by damaging the outer membrane and simultaneously enhancing membrane potential [28]. However, that study did not address the apparent contradiction between outer membrane disruption and increased membrane potential, underscoring the need for further mechanistic clarification.

## 5. Conclusions

This study demonstrates that metformin significantly enhances the antibacterial activity of doxycycline against *Pm*, particularly serogroup A strains. Building upon previous findings, we showed that this combination not only exhibits strong in vitro synergy but also improves survival and reduces bacterial burden in both mammalian and avian infection models, with minimal toxicity. Histopathological analysis further confirmed its protective effects on tissue integrity. Mechanistically, metformin enhances the antibacterial activity of doxycycline against *Pm* by increasing membrane permeability, dissipating membrane potential, and promoting intracellular antibiotic accumulation. Morphological evidence of intracellular content leakage and structural instability further supports this synergistic mechanism. These findings highlight metformin as a promising tetracycline adjuvant for veterinary treatment of *Pm* infections, while underscoring the need for further studies on host interactions and long-term safety.

## Data Availability

The raw data supporting the conclusions of this article will be made available by the authors on request.

## Supplementary Materials

The following supporting information can be downloaded at: https://www.mdpi.com/article/10.3390/microorganisms13081724/s1, Table S1: Detailed information of all *Pm* strains used in this study; Table S2: MICs and the resistance genes profile of FCF83 strain; Table S3: Antibiotic and non-antibiotic compounds used in this study; Figure S1: Checkerboard and time–kill analyses evaluating the synergistic antibacterial activity of metformin combined with various antibiotics against FCF83; Figure S2: Safety evaluation and resistance development analysis of doxycycline–metformin combination.

## Abbreviations

The following abbreviations are used in this manuscript:

*Pm*	*Pasteurella multocida*
*PmA*	*Pasteurella multocida* serotype A
LPS	Lipopolysaccharide
NPN	1-N-phenylnaphthylamine
PI	propidium iodide
DiSC_3_(5)	3,3-dipropylthiadicarbocyanine iodide
BCECF-AM	2′,7′-Bis-(2-carboxyethyl)-5-(and-6)-carboxyfluorescein acetoxymethyl ester
MLST	Multi-locus sequence typing
FDA	U.S. Food and Drug Administration
PMF	Proton motive force
TSA	Tryptic soy agar
TSB	Tryptic soy broth
FBS	Fetal bovine serum
CHO	Chinese hamster ovary
DMEM	Dulbecco’s Modified Eagle’s Medium
MIC	Minimum inhibitory concentration
CA-MHB	Cation-adjusted Mueller–Hinton broth
OD	Optical density
FIC	Fractional inhibitory concentration
FICI	FIC index
PBS	Phosphate-buffered saline
CFU	Colony-forming unit
HEPES	4-(2-hydroxyethyl)-1-piperazineethanesulfonic acid
TEM	Transmission electron microscopy
SEM	Scanning electron microscopy
cDNA	Complementary DNA
qRT-PCR	Quantitative real-time PCR
DOX	Doxycycline
MET	Metformin
