# Supplementary material for: Metformin Enhances Doxycycline Efficacy Against Pasteurella multocida: Evidence from In Vitro, In Vivo, and Morphological Studies
Source: Microorganisms. 2025 Jul 23;13(8):1724. doi: 10.3390/microorganisms13081724 (PMC12388482; doi:10.3390/microorganisms13081724)
Supplement: Supplementary file 1 [file microorganisms-13-01724-s001.zip › microorganisms-3698883-supplementary.pdf]

Table S1. Detailed Information of All *Pm* Strains Used in This Study

Strain	Capsular serogroup: LPS: MLST	Origin	GenBank/SRA accession No.	MIC of Doxycycline (μg/mL)
FCF83	A: L1: ST129	Duck	NZ_CP038875	16
FCF12	D: L6: ST50	Pig	SRR25224598	2
FCF79	F: L3: ST176	Monkey	SRR25224629	1
FCF147	-: L2: -	Swan	NZ_CP143490	1

Table S2. MICs and the Resistance Genes Profile of FCF83 strain

Antibiotic class	Resistant gene	Antimicrobial agent	MIC (μg/mL)
β-lactams	$bla_{\text{OXA-1}}$	Ampicillin	2
		Amoxicillin	4
Aminoglycosides	*aph*(3')-*Ia*,	Kanamycin	32
	ant(2")-*Ia*,	Gentamycin	16
	strA，*strB*	Streptomycin	64
Macrolides	*mef*(B)	Erythromycin	8
		Tilmicosin	4
Phenicols	*cat*(P)	Chloramphenicol	32
		Florfenicol	0.5
Tetracyclines	*tet*(B)	Tetracycline	32
		Doxycycline	16
Sulfonamides	sul2, sul3	Sulfadiazine	256
Fluoroquinolones	-	Ciprofloxacin	1
		Enrofloxacin	0.5

Table S3. Antibiotic and Non-antibiotic Compounds Used in This Study

Antibiotics/adjuvants	Antibiotic class	Solvent used	Note
Doxycycline	Tetracyclines	Sterile distilled water	Light-sensitive
Tetracycline	Tetracyclines	Sterile distilled water	Light-sensitive
Kanamycin	Aminoglycosides	Sterile distilled water	-
Streptomycin	Aminoglycosides	Sterile distilled water	-
Erythromycin	Macrolides	95% ethanol	Light-sensitive
Ampicillin	β-lactams	Sterile distilled water	-
Chloramphenicol	Phenicols	Ethanol or DMSO	-
Sulfadiazine	Sulfonamides	Ethanol or DMSO	-
Ciprofloxacin	Fluoroquinolones	Sterile distilled water	Adjust pH to improve solubility
Metformin	-	Sterile distilled water	-
Carprofen	-	Ethanol or DMSO	-
Benzydamine	-	Sterile distilled water	Heat-sensitive
Benserazide	-	Sterile distilled water	Acidic pH to improves solubility
Loperamide	-	DMSO	-

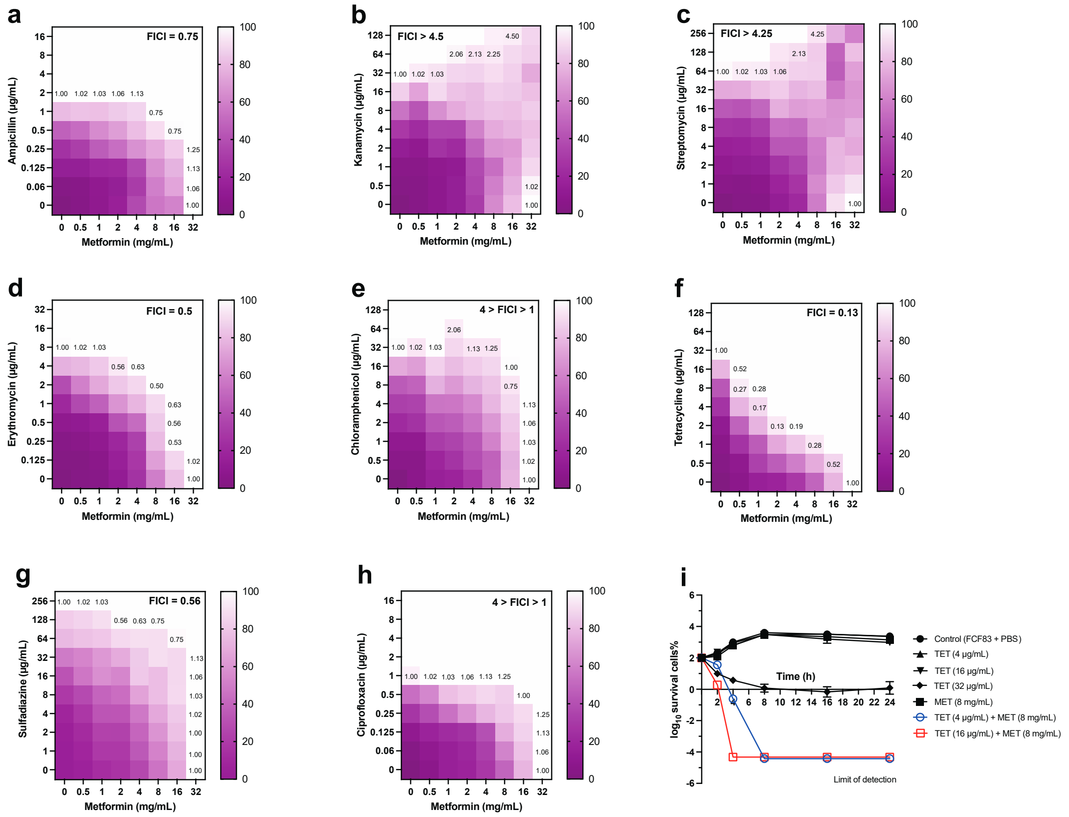

Figure S1. Checkerboard and time-kill analyses evaluating the synergistic antibacterial activity of metformin combined with various antibiotics against FCF83. (a–h) Inhibition heatmaps and FICI values for metformin (0–32 mg/mL) in combination with ampicillin, kanamycin, streptomycin, erythromycin, chloramphenicol, tetracycline, sulfadiazine, and ciprofloxacin. Gradient bars indicate the percentage of bacterial growth inhibition. Synergistic effects (FICI ≤ 0.5) were observed with doxycycline, tetracycline, and erythromycin, whereas combinations with ampicillin, chloramphenicol, ciprofloxacin, and sulfadiazine showed partial or no synergy. Antagonistic interactions (FICI > 4) were found with streptomycin and kanamycin. (i) Time-kill curves showing enhanced bactericidal activity when combined with metformin (MET, 8 mg/mL) at varying tetracycline (TET) concentrations (4, 16, and 32 μg/mL), compared to tetracycline alone or control. Data represent the means of three independent experiments.

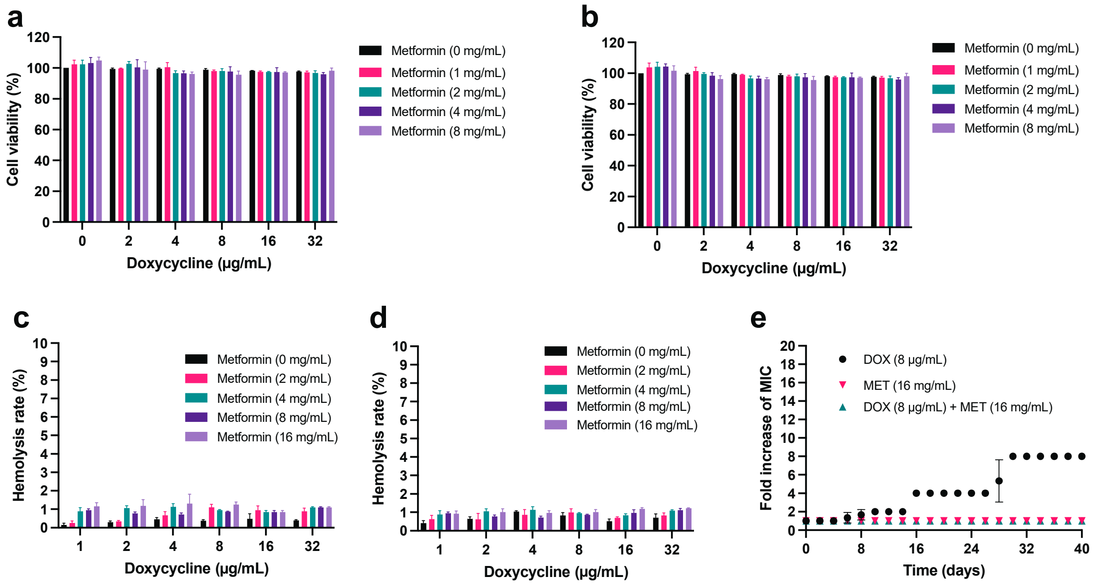

Figure S2. Safety evaluation and resistance development analysis of doxycycline–metformin combination. (a–b) Cytotoxicity of doxycycline alone or in combination with increasing concentrations of metformin (0–8 mg/mL) was evaluated in CHO (a) and DF-1 (b) cells after 24 h of co-incubation using a CCK-8 assay. Cell viability remained above 97% across all treatment conditions. (c–d) Hemolytic activity of doxycycline combined with varying concentrations of metformin (0–16 mg/mL) was assessed using rabbit (c) and chicken (d) erythrocytes. Hemolysis rates remained below 2% under all conditions, indicating low hemolytic toxicity. (e) Resistance development assay for *Pm* strain FCF83 under daily exposure to doxycycline (8 μg/mL), metformin (16 mg/mL), or their combination over 40 days. MIC fold changes relative to the initial MIC were recorded. The combination treatment significantly delayed the emergence of resistance compared with doxycycline alone. Data represent mean ± SD from three independent experiments.
